# Editorial: Targeted therapies in gastric cancer: molecular signatures and immune microenvironment insights

**DOI:** 10.3389/fimmu.2026.2013952

**Published:** 2026-10-27

**Authors:** Alessandro Mangogna, Laura Mariuzzi, Giuseppe Aprile, Matteo Fassan, Massimo Milione

**Affiliations:** 1 Institute of Pathologic Anatomy, Department of Medicina (DMED), University of Udine, Udine, Italy; 2 Department of Medical Oncology, University Hospital of Udine, Udine, Italy; 3 Department of Medicine (DIMED), University of Padua, Padua, Italy; 4 Veneto Institute of Oncology (IOV-IRCCS), Padua, Italy

**Keywords:** biomarkers, gastric cancer, precision oncology, targeted therapy, tumor microenvironment

## 1 Introduction

Gastric cancer remains a major cause of cancer-related mortality and illustrates why an anatomically defined malignancy cannot be treated as a single biological entity (1, 2). Histological diversity, divergent etiologic pathways, genomic instability, epigenetic remodeling, metabolic plasticity, and marked spatial and temporal heterogeneity of the immune microenvironment all contribute to variable clinical behavior and therapeutic response (3–5). The introduction of HER2-directed treatment, immune-checkpoint inhibition, antiangiogenic agents, and, more recently, therapies targeting claudin 18.2 (CLDN18.2) has expanded treatment options (6–9), which are all supported by the results of randomized phase III trials. Yet these advances have also exposed a central limitation of biomarker-driven oncology: a single marker measured at a single time point rarely captures the evolving biology of an individual tumor (10).

The nineteen contributions in this Research Topic address this limitation across gastric carcinogenesis, biomarker discovery, immune profiling, treatment selection, and clinical response. Together, they define a progression from etiologic understanding and multimodal profiling to integrated stratification, treatment selection, and longitudinal reassessment (**Figure 1**). Their collective message is that the next advance will not come from adding another prognostic signature, but from linking analytically validated biomarkers to spatially and temporally defined biological states that can guide treatment.

**Figure 1:**
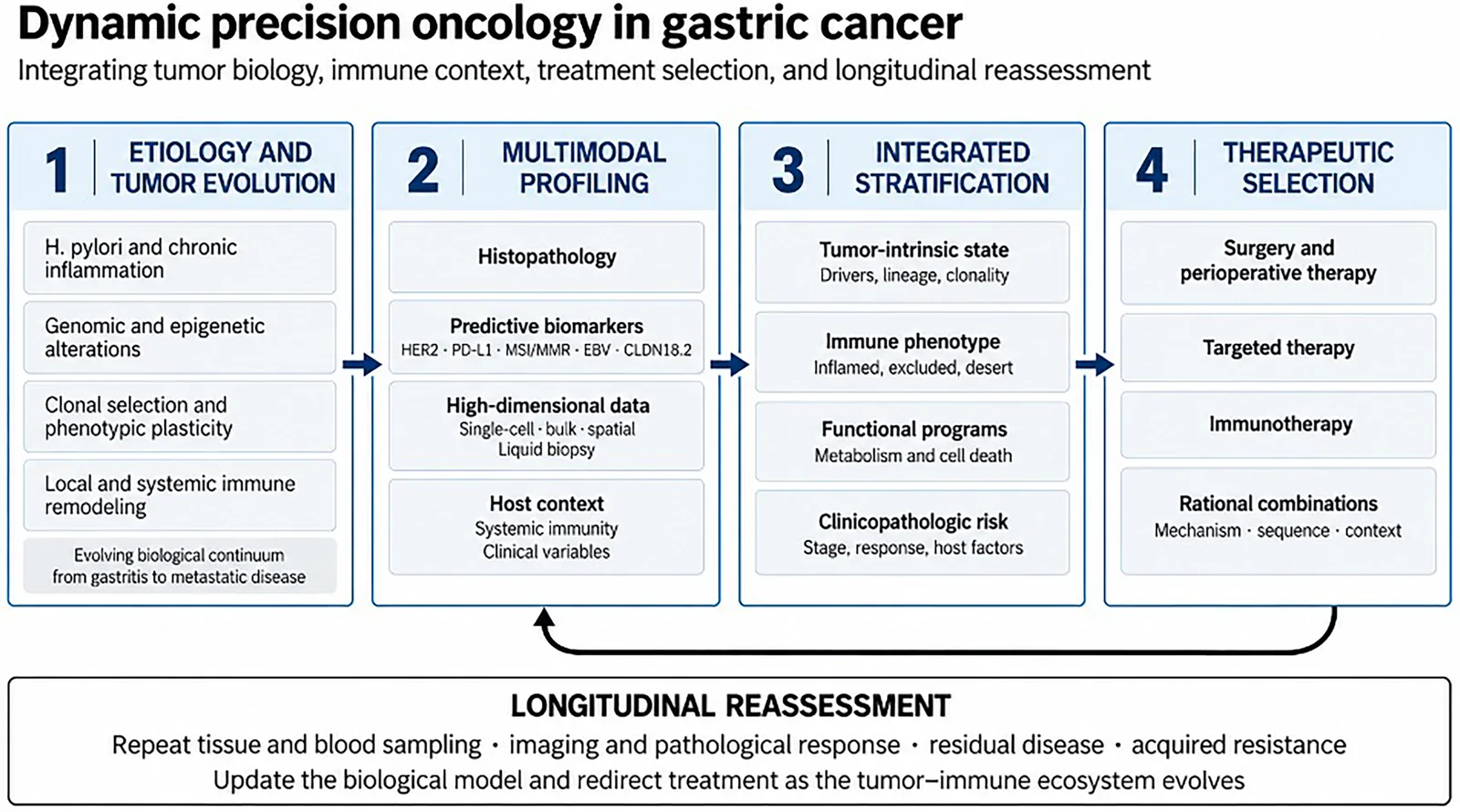
From multidimensional profiling to dynamic precision oncology in gastric cancer. Etiologic exposures and molecular alterations drive tumor evolution. Histopathology, predictive biomarkers, high-dimensional molecular profiling, and immune context assessment together enable integrated patient stratification. This approach guides the selection of surgery, perioperative treatment, targeted therapy, immunotherapy, or their combinations. Ongoing reassessment using tissue and blood samples, imaging, and pathological response updates the biological model as residual disease and resistance develop. CLDN18.2, claudin 18.2; EBV, Epstein–Barr virus; HER2, human epidermal growth factor receptor 2; MMR, mismatch repair; MSI, microsatellite instability; PD-L1, programmed death-ligand 1.

## 2 Tracing the biological continuum of gastric cancer

Several contributions place gastric cancer in its etiologic and evolutionary context. Song et al. map immune trajectories from *Helicobacter pylori* gastritis to gastric cancer, emphasizing the interaction of epithelial injury, microbial stimuli, chronic inflammation, and immune remodeling. Li et al. use weighted gene co-expression network analysis to identify modules and candidate prognostic markers associated with malignant transformation. These studies suggest that markers of transition may complement conventional detection and risk-stratification strategies.

The search for clinically meaningful biomarkers is also examined at a field-wide level. Komekbay et al. map trends in gastric cancer tumor-marker research from 2019 to 2024, whereas Cai et al. identify calcium-signaling-related biomarkers associated with lymph-node metastasis in T1 and T2 tumors. Together, these studies show the breadth of biomarker discovery and the need to link molecular associations with defined pathological endpoints.

## 3 Decoding the tumor and immune ecosystems

A major strength of the collection is its emphasis on the tumor microenvironment as an active determinant of progression and therapeutic vulnerability. Yang et al. integrate single-cell and bulk transcriptomic data to examine IL1RAP in relation to the gastric cancer microenvironment and prognosis. Zhang et al. investigate IL-36-related genes as prognostic indicators. Wang et al. identify HIGD1B as a candidate biomarker linked to immune-cell infiltration and adverse outcome. Although these studies nominate different molecular nodes, they converge on a shared principle: the prognostic effect of a tumor-associated gene cannot be separated from the cellular ecosystem in which it is expressed.

The relevant immune ecosystem extends beyond the primary tumor. Mano et al. characterize immune cells in omental milky spots, a niche relevant to peritoneal dissemination, while Zhang et al. review systemic immune dysregulation and its potential clinical applications. Tissue-based analysis provides spatial and cellular resolution; circulating parameters may provide repeatable, minimally invasive measurements of host-tumor interactions.

The meta-analysis by Tan et al. evaluates the prognostic value of neutrophil-to-lymphocyte, platelet-to-lymphocyte, and lymphocyte-to-monocyte ratios in patients receiving immune-checkpoint inhibitors. These readily obtainable markers are attractive when repeated tissue sampling is impractical. However, their clinical simplicity must be balanced against limited biological specificity and sensitivity to infection, comorbidity, medication, and treatment timing. Their most promising role may lie in multivariable models rather than alone.

## 4 Metabolism, hypoxia, and regulated cell death

The Research Topic further highlights biological programs that bridge tumor-cell fitness and immune regulation. Li et al. develop a prognostic model that integrates hypoxia and mitochondrial dysfunction using single-cell and bulk RNA-sequencing analyses. Chen et al. investigate polyamine-metabolism biomarkers and their relationship with immune features and outcome. Liu et al. integrate parthanatos-related genes with microenvironmental characteristics using a machine-learning framework. Song et al. analyze multiple programmed cell-death patterns to predict prognosis and drug sensitivity. Considered together, these investigations suggest that metabolism, cellular stress, and regulated cell death are not merely downstream manifestations of aggressive disease. They may shape antigenicity, cytokine signaling, immune recruitment, and treatment sensitivity, thereby providing a functional layer that complements genomic classification.

These studies also expose a translational checkpoint. Models derived from public or retrospective datasets require rigorous control of cohort composition, platform effects, endpoint definitions, and overfitting. Before clinical use, signature need independent multicenter validation, comparison with established clinicopathological variables, and evidence of incremental clinical utility. Spatial validation is especially important because bulk measurements may conflate tumor, stromal, and immune-cell signals.

## 5 From signatures to therapeutic decisions

Tian et al. combine functional and clinical validation to define transfer-RNA-derived small-RNA molecular subtypes with potential implications for precision treatment. This work exemplifies the movement from descriptive omics to biologically anchored stratification. At the clinical interface, Wang et al. construct a nomogram that incorporates immune-related adverse events and clinical characteristics to predict survival in patients with advanced gastric or gastroesophageal-junction adenocarcinoma receiving anti-PD-1 therapy. Immune-related adverse events may reflect pharmacodynamic immune activation. However, their time-dependent nature and the possibility of guarantee-time bias require careful modeling before using them prognostically.

Clinical translation is represented at different evidence levels. Hu et al. describe a patient with resectable locally advanced gastric cancer treated with SOX chemotherapy, apatinib, and camrelizumab, a hypothesis-generating example of combined cytotoxic, antiangiogenic, and immune treatment. Liu et al. provide real-world evidence on pathological response and safety across preoperative regimens followed by D2 gastrectomy. Standardized tumor-regression grading, comprehensive sampling, and correlation with long-term survival are needed to interpret pathological response.

Yuan et al. review multimodal strategies, including approaches that directly or indirectly modify the tumor microenvironment. Together, these studies support selecting combinations according to mechanism, treatment sequence, and biomarker context rather than simply increasing treatment intensity.

## 6 Heterogeneity, resistance, and the problem of time

Heterogeneity in gastric cancer is interpatient, intratumoral, intermetastatic, and temporal. Biomarker-positive and biomarker-negative clones may coexist within one lesion, and metastases may diverge from the primary tumor. Small biopsies may therefore underrepresent target expression, immune-cell distribution, or regional hypoxia, whereas resection specimens provide broader spatial information but often reflect an earlier disease state.

Therapy can select resistant subclones and alter antigen presentation, stromal composition, immune populations, metabolism, and regulated cell-death programs (Li et al., Chen et al., Liu et al., Song et al.). The relevant question is therefore whether a target remains present, accessible, and functionally important when treatment is chosen. This supports reassessment at progression by repeat tissue biopsy when feasible, complemented by liquid biopsy or imaging when it is not.

Resistance is also an ecosystem-level phenomenon. Immune exclusion, T-cell dysfunction, suppressive myeloid populations, fibroblast-mediated barriers, vascular abnormalities, and nutrient competition may reduce efficacy despite favorable tumor-cell biomarkers. Findings from omental immune niches (Mano et al.), systemic immunity (Zhang et al.), and circulating inflammatory indices (Tan et al.) support assessment across anatomical and biological compartments.

## 7 From discovery signatures to clinically useful tests

Statistical prediction does not establish clinical utility. Single-cell sequencing, bulk transcriptomics, co-expression networks, and machine learning reveal relationships inaccessible to conventional assays (Tian et al., Li et al., Chen et al., Liu et al., Song et al., Li et al., Yang et al.), but retrospective discrimination alone does not show that a signature improves care. A model must remain accurate across populations, stages, specimen types, platforms, and treatment contexts and add information beyond routine variables.

Translation requires biological and orthogonal validation, an analytically reproducible assay, robustness to pre-analytical variation, and a clinically realistic turnaround time. Cutoffs and intended-use populations should be prespecified before external validation. Prospective studies must then determine whether testing changes treatment selection, improves outcomes, or avoids ineffective therapy.

The nineteen contributions span different positions along this continuum, from mechanistic and computational discovery to tissue- and blood-based measurement, therapeutic reviews, and clinical reports. The next step is to connect these layers in prospective, biomarker-rich trials that analyze pathology, molecular data, immune context, treatment exposure, response, and survival in the same patients.

## 8 Toward dynamic, integrated biomarkers

Across these nineteen articles, four priorities emerge. Biomarkers should be composite, integrating morphology, stage, established predictive markers, molecular signatures, immune composition, and systemic inflammatory indices. They should be spatially informed because primary tumors, invasive fronts, lymph-node metastases, omental niches, and distant deposits may differ. They should also be longitudinal because treatment changes clonal composition, target expression, metabolism, and immune context. Finally, they require analytical reproducibility, prespecified cutoffs, independent validation, and evidence that testing changes management.

The pathologist has a central role in this integration. Morphology provides the spatial framework where molecular and immune signals acquire meaning. Adequate sampling and quality control determine the reliability of downstream assays. Digital pathology, multiplex imaging, spatial transcriptomics, and artificial-intelligence-assisted quantification offer opportunities to measure heterogeneity more objectively. These methods should augment rather than detach from clinicopathological interpretation and must be developed with transparent algorithms and external validation.

## 9 Conclusions

This Research Topic reflects the transition from a limited set of static, treatment-linked biomarkers to multidimensional models of gastric cancer. Across the contributions, the same translational sequence emerges: define etiologic and biological context, profile the tumor and host, convert these measurements into clinically meaningful strata, select appropriate upfront treatment strategy, and reassess the disease as it evolves, in order to adapt or refine the therapeutic opportunities.

The field does not need an expanding catalogue of prognostic signatures. It needs a smaller number of reproducible biomarkers that identify actionable biological states, remain informative across disease evolution, and improve treatment decisions. Achieving this goal will require pathology, molecular profiling, immune assessment, and longitudinal sampling to be developed as parts of the same clinical framework.
